# Metagenomic approach for the isolation of a thermostable β-galactosidase with high tolerance of galactose and glucose from soil samples of Turpan Basin

**DOI:** 10.1186/1471-2180-13-237

**Published:** 2013-10-24

**Authors:** Xia Zhang, He Li, Chang-Jie Li, Teng Ma, Gang Li, Yu-Huan Liu

**Affiliations:** 1 School of life sciences, Sun Yat-sen University, Guangzhou 510275, People’s Republic of China; 2 Guangdong Provincial Key Laboratory of Pharmaceutical Bioactive Substances, School of Basic Courses, Guangdong Pharmaceutical University, Guangzhou 510006, People’s Republic of China

**Keywords:** β-Galactosidases, Metagenomic library, Enzyme characterization, Thermostability, Tolerance of galactose and glucose

## Abstract

**Background:**

β-Galactosidases can be used to produce low-lactose milk and dairy products for lactose intolerant people. Although commercial β-galactosidases have outstanding lactose hydrolysis ability, their thermostability is low, and reaction products have strong inhibition to these enzymes. In addition, the β-galactosidases possessing simultaneously high thermostability and tolerance of galactose and glucose are still seldom reported until now. Therefore, identification of novel β-galactosidases with high thermostability and tolerance to reaction products from unculturable microorganisms accounting for over 99% of microorganisms in the environment via metagenomic strategy is still urgently in demand.

**Results:**

In the present study, a novel β-galactosidase (Gal308) consisting of 658 amino acids was identified from a metagenomic library from soil samples of Turpan Basin in China by functional screening. After being overexpressed in *Escherichia coli* and purified to homogeneity, the enzymatic properties of Gal308 with N-terminal fusion tag were investigated. The recombinant enzyme displayed a pH optimum of 6.8 and a temperature optimum of 78°C, and was considerably stable in the temperature range of 40°C - 70°C with almost unchangeable activity after incubation for 60 min. Furthermore, Gal308 displayed a very high tolerance of galactose and glucose, with the highest inhibition constant *K*_i,gal_ (238 mM) and *K*_i,glu_ (1725 mM) among β-galactosidases. In addition, Gal308 also exhibited high enzymatic activity for its synthetic substrate *o*-nitrophenyl-β-D-galactopyranoside (ONPG, 185 U/mg) and natural substrate lactose (47.6 U/mg).

**Conclusion:**

This study will enrich the source of β-galactosidases, and attract some attentions to β-galactosidases from extreme habitats and metagenomic library. Furthermore, the recombinant Gal308 fused with 156 amino acids exhibits many novel properties including high activity and thermostability at high temperatures, the pH optimum of 6.8, high enzyme activity for lactose, as well as high tolerance of galactose and glucose. These properties make it a good candidate in the production of low-lactose milk and dairy products after further study.

## Background

β-Galactosidases (EC 3.2.1.23), which hydrolyze lactose to glucose and galactose, have two main applications in food industry, including production of low-lactose milk and dairy products for lactose intolerant people and production of galacto-oligosaccharides from lactose by the transgalactosylation reaction [1]. Traditionally, commercial β-galactosidases are produced from fungi of the genus *Aspergillus* and yeasts of the genus *Kluyveromyces*[2]. Despite these β-galactosidases have outstanding lactose hydrolysis ability, they have two major drawbacks including low thermostability and high inhibition of reaction products. Commonly, the optimum termperatures of these enzymes are less than 58°C [3,4], and thus they have low stability during the high-temperature (65–85°C) pasteurization of milk. Furthermore, these enzymes are badly inhibited in the presence of the reaction products (galactose and glucose) [5,6], and the inhibition of reaction products may lead a decrease in the reaction rates or even stop enzymatic reaction completely. These two problems can be solved using thermostable β-galactosidases with high tolerance of galactose and glucose. Therefore, interests in identifying novel β-galactosidases with high thermostablility or high tolerance of galactose and glucose have been increasing in the last decade. Despite some thermostable β-galactosidases have been found from thermophilic microorganisms [7-13], and several β-galactosidases from mesophilic microorganisms with high tolerance of galactose or glucose have also been identified [13-15], the β-galactosidases possessing simultaneously high thermostablity and tolerance of galactose and glucose are still seldom reported until now. Furthermore, almost all of reported β-galactosidases are from cultured microorganisms, and little attention has been paid to β-galactosidases from unculturable microorganisms, which account for over 99% of microorganisms in the environment [16]. Therefore, some efforts should be made to discover novel β-galactosidases with high thermostability and tolerance to reaction products from unculturable microorganisms of environment.

To discover novel biocatalyst from uncultured microorganisms in the environment, the metagenomic approach has been successfully employed in the isolation and identification of novel enzymes [17]. However, there are few reports on β-galactosidases obtained via metagenomic strategies up to now. Recently, a novel β-galactosidase gene, *zd410*, was isolated by screening a soil metagenomic library [18]. Nevertheless, this enzyme was regarded as a cold-adapted β-galactosidase due to its optimal temperature of 38°C and 54% residual activity at 20°C. Thus, identification of novel β-galactosidases with high thermostability and low inhibition of reaction product via metagenomic strategy is still urgently in demand.

In the present study, a metagenomic library from soil samples of Turpan Basin, the hottest and driest area in China, was constructed, and a novel β-galactosidase (Gal308) was identified and expressed in *Escherichia coli* (*E. coli*). The enzymatic properties of Gal308 with N-terminal fusion tag were investigated after purification, and this enzyme displayed several novel properties including high thermostability, high tolerance of galactose and glucose, as well as high enzymatic activity for lactose. These properties make it a good candidate in the production of low-lactose milk and dairy products after further study.

## Results

### Screening for β-galactosidase from a metagenomic library

**Figure 1:**
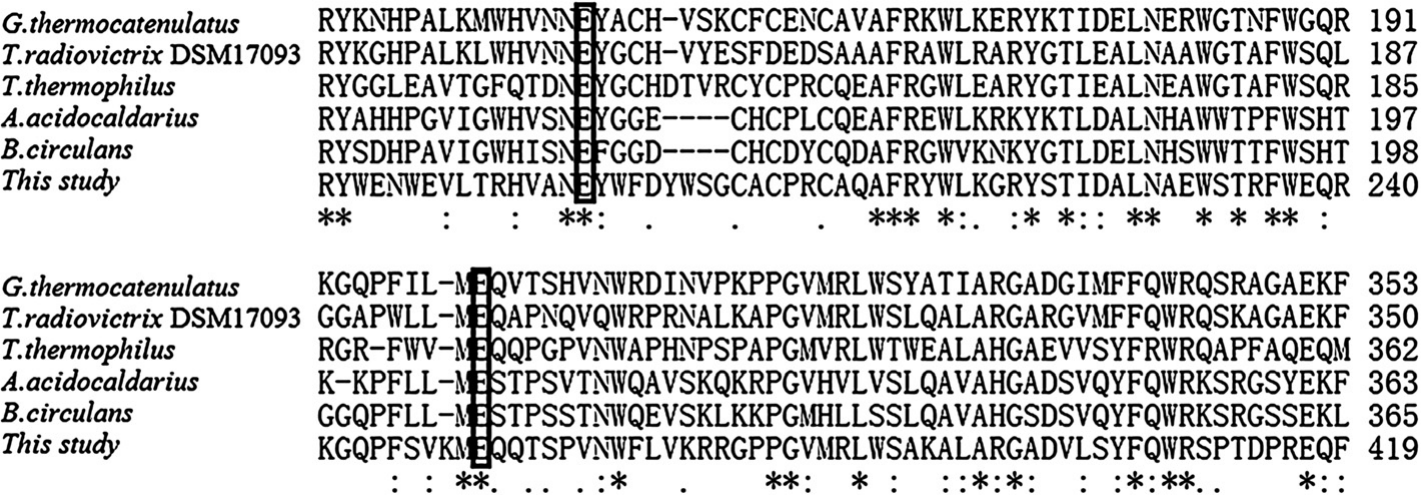
**Identification of the active site residues of Gal308 by alignment of the amino acid residues with other five homologous β-galactosidases from GH family 42.** The GenBank accession numbers are as follows: *Geobacillus thermocatenulatus*, AAW56416; *Truepera radiovictrix* DSM17093, ADI14846; *Thermus thermophilus*, ABI35985; *Alicyclobacillus acidocaldarius*, AAZ81841; *Bacillus circulans*, AAA22260; This study (Gal308), AFD21844. The alignment was carried out using the Clustal W method. The number flanking the sequences represents amino acid positions of each sequence. *Asterisks* mean identity. The two putative catalytic residues (E195 and E368) of Gal308 were shown in box.

To discover novel thermostable β-galactosidases, a metagenomic library consisting of approximately 8,000 clones was constructed using DNA extracted from soil samples of the Mountain of Flames of the Turpan Basin in China. Restriction analysis of 20 randomly selected clones from metagenomic library indicated that 95% of clones contained inserts of 2.5 to 7.5 kb in size, with an average size of 4.2 kb. Thus, the metagenomic library covered theoretically about 33.6 MB of soil microbial community DNA. One positive clone with bright blue color was finally identified from the metagenomic library. The activity of the positive clone was reconfirmed after retransformation, and then the plasmid of this clone was extracted and an insert of 5215 bp was sequenced. The ORF-finder and blastX analysis revealed the presence of an open reading frame of 1977 bp, which encoded a glycoside hydrolase family 42 (GH family 42) protein (Gal308) of 658 amino acids. A protein blast (blastp) search in the databases of NCBI indicated that the protein had the highest identity of 49% (291/599) with the β-galactosidase from one thermophilic microbe *Geobacillus thermocatenulatus*, as well as a low identity of only 38% (224/593) with the β-galactosidase from the other thermophilic microbe *Thermoanaerobacterium thermosaccharolyticum* DSM 571, suggesting that Gal308 is probably a novel thermostable β-galactosidase from unculturable microorganisms. In addition, multiple sequence alignment of Gal308 and other five homologous β-galactosidases from GH family 42 allowed the identification of the active site residues of Gal308 (Figure 1). Glu141 (E141) and Glu312 (E312) were regarded as the catalytic residues of a typical GH-42 β-galactosidase [19]. Thus, E195 and E368 (marked with two boxes), which located in two conserved regions, were thought to be the active site residues of Gal308 based on amino acid sequence alignment and the determined structure of β-galactosidase from *T. Thermophilus* (Figure 1).

### Heterologous expression and purification of recombinant Gal308

**Figure 2:**
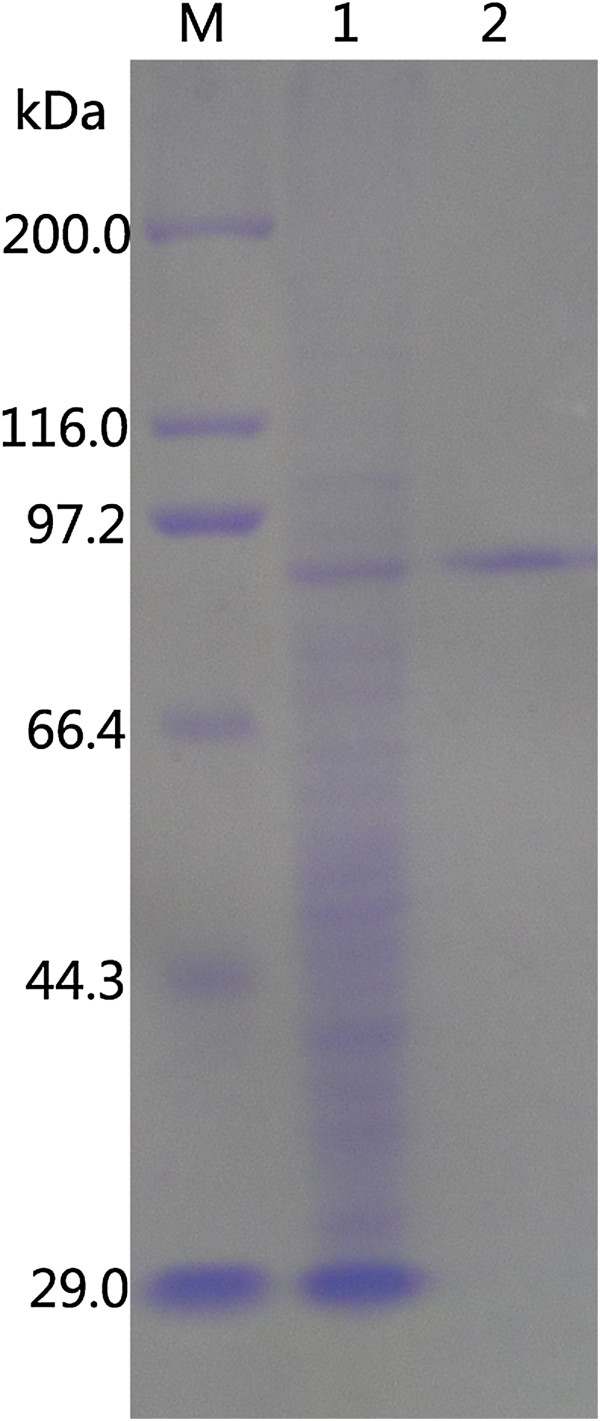
**SDS-PAGE analysis of recombinant Gal308 from supernatant of *E. coli *BL21 (DE3) cell lysates and purified Gal308 by affinity chromatography.** Lanes: M, standard protein molecular mass markers (sizes in kilodaltons are indicated on the left); 1, recombinant Gal308 from supernatant of *E. coli* BL21 (DE3) cell lysates; 2, recombinant Gal308 purified by His•Bind® Purification Kit. The sizes in kilodaltons of protein marker were listed as follows: porcine heart myosin (200,000 Da), *E. coli* β-galactosidase (116,000 Da), rabbit muscle phosphorylase B (97,200 Da), bovine serum albumin (66,409 Da), ovalbumin (44,287 Da), carbonic anhydrase (29,000 Da).

To investigate the biochemical properties of Gal308, *E. coli* expression vector pET-32a(+) was used to express recombinant protein under the conditions described in materials and methods. The cells were harvested and disrupted by sonication in ice-water bath. The cell lysate was found fully clear, and no inclusion bodies were formed, which suggested that the recombinant Gal308 was highly soluble. Then, the recombinant Lac308 with a six-histidine tag was purified by Ni-NTA chromatography, and the result showed that Ni-NTA chromatography of cell lysate led to 6.25-fold purification and 85% activity yield (Table 1). Furthermore, the purified enzyme and the crude enzyme (supernatant from cell lysates) were applied to SDS-PAGE (Figure 2) together to determine the molecular mass and expression level of recombinant protein. The purified recombinant protein showed a single protein band of approximate 95 kDa, higher than its calculated molecular mass (76.77 kDa), which can be ascribed to its N-terminal fusion of 156 amino acids (about 18 kDa) corresponding to thioredoxin tag (Trx·Tag), polyhistidine tag (His·Tag), S·Tag epitope (S·Tag), and a unique thrombin cleavage site (thrombin). In addition, the highest expression level of *gal308* in *E. coli* was about 125 mg/L when the cell was induced at 30°C for 8 h. Next, the purified Gal308 was used to study its biochemical properties.

**Table 1:** Purification of Gal308

**Purification step**	**Total protein (mg)**	**Total activity (U)**	**Specific activity (U/mg)**	**Fold purification**	**Activity yield (%)**
Cell lysate	37.94	1122.21	29.58	1.00	100.00%
Ni-NTA chromatography	5.16	953.88	184.86	6.25	85.00%

### Effect of pH and temperature on enzymatic activity and stability

**Figure 3:**
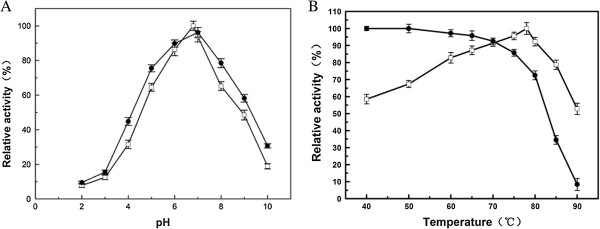
**Effect of pH (A) and temperature (B) on activity (*square*) and stability(*circle*) of Gal308 using lactose as substrate.** Data points are the average of triplicate measurements; *error bars* represent ±1 SD.

The optimal pH of recombinant Gal308 was investigated by measuring the enzymatic activity towards lactose at various pH values (pH 2.0-10.0) and 78°C. Gal308 displayed the highest activity at pH 6.8. Even at pH 4.0 and pH 10.0, recombinant enzyme still exhibited 31.6% and 18.9% of the maximum activity, respectively (Figure 3A). Moreover, the enzyme was found to be stable in the pH range of 5.0 - 8.0, and more than 70% of the maximum activity was remained (Figure 3A). Thus, the pH properties of Gal308 are suitable in lactose hydrolysis of natural milk (pH 6.7-6.8). The optimal temperature for the enzyme was 78°C (Figure 3B). The thermostability of Gal308 was drastically decreased when the temperature was more than 80°C, and the enzyme was almost completely inactivated at 90°C (Figure 3B). However, the enzyme was fairly stable for a temperature range of 40°C - 70°C, and its activity almost kept unchangeable after incubation for 60 min. Therefore, Gal308 is especially suitable for hydrolysis of lactose during milk pasteurization (62.8°C - 65.6°C for 30 min) when compared with a commercially available β-galactosidase from *Kluyveromyces lactis* (the optimal temperature is approximately 50°C).

### The effect of metal ions on enzymatic activity

Following the addition of Na^+^, K^+^, Mn^2+^ and Zn^2+^, no pronounced effect on the enzymatic activity was observed. However, the presence of 1 mM Cu^2+^, Fe^3+^, and Al^3+^ caused a strong inhibition to the enzymatic activity. In addition, the existence of 1 mM Mg^2+^ and Ca^2+^ slightly stimulated the enzymatic activity.

### Substrate specificity and kinetic parameters

The substrate specificity of Gal308 towards several chromogenic nitrophenyl analogues and its natural substrate lactose was shown in Table 2. The enzyme displayed high hydrolysis ability for ONPG (100%) and moderate activity for its natural substrate lactose (25.7%). However, the hydrolysis ability of the enzyme towards all other chromogenic nitrophenyl analogues was very weak, indicating that Gal308 is a β-galactosidase with narrow substrate specificity. To investigate the kinetic parameters of recombinant enzyme, the Michaelis-Menten constants (*K*_m_), turnover numbers (*k*_cat_), and catalytic efficiencies (*k*_cat_/*K*_m_) of Gal308 for ONPG and lactose were determined. The *k*_cat_ and *K*_m_ values were 464.7 ± 7.8 s^-1^ and 2.7 ± 0.3 mM for ONPG, and 264.2 ± 2.1 s^-1^ and 7.1 ± 0.8 mM for lactose, respectively. The *k*_cat_/*K*_m_ value of the enzyme for ONPG (172.1 s^-1^ mM^-1^) was 4.6-fold higher than that for lactose (37.2 s^-1^ mM^-1^), which clearly demonstrated that the catalytic efficiency of Gal308 for ONPG was much higher than that for lactose.

**Table 2:** Relative activity of purified Gal308 with several nitrophenyl-derived chromogenic substrates and its natural substrate lactose

**Substrate**	**Activity**^***a ***^**(%)**
*o*-Nitrophenyl-β-D-galactopyranoside (ONPG)	100
*p*-Nitrophenyl-β-D-galactopyranoside (pNPG)	<1
*o*-Nitrophenyl-β-D-fucopyranoside	<1
*p*-Nitrophenyl-β-D-mannoside	3.5±0.3
*o*-Nitrophenyl-β-D-glucoside	<1
*p*-Nitrophenyl-β-D-xyloside	5.7±0.2
*p*-Nitrophenyl-β-D-cellobioside	<1
*p*-Nitrophenyl-β-D-lactoside	7.8±0.3
*p*-Nitrophenyl-α-D-galactoside	<1
Lactose	25.7±1.8

^*a*^ The values are relative to the 100% value observed with ONPG (185 U/mg).

Hydrolysis of lactose in milk by Gal308.

### Effects of galactose and glucose on the activity of Gal308

**Figure 4:**
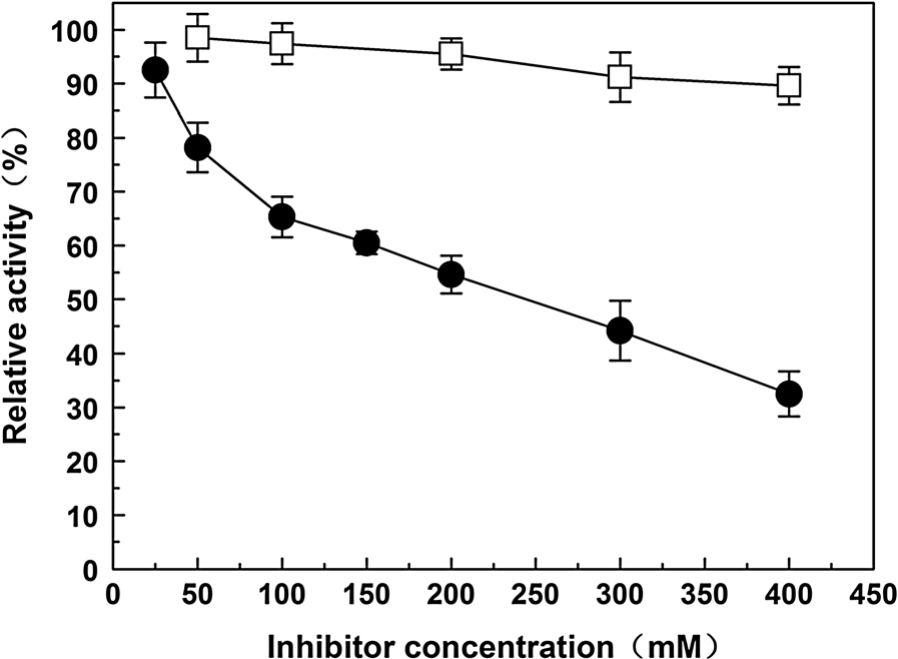
**Effects of galactose (*circle*) and glucose (*square*) as inhibitors on the activity of Gal308.** The reactions were performed under standard conditions with ONPG as a substrate. The relative activity was defined as the relative value to the maximum activity without galactose or glucose. Data represent the means of three experiments and error bars represent standard deviation.

Lineweaver-Burk plots (1/V vs. 1/[S]) were used to investigate the effects of the inhibitors galactose and glucose on the activity of Gal308 using ONPG as substrate. The results demonstrated that both of galactose and glucose were competitive inhibitors of Gal308 because *V*_max_ value of Gal308 was unchangeable and *K*_m_ value of Gal308 was increased with concentration enhancement of the inhibitors (data not shown). Furthermore, the inhibition constant (*K*_i_) of galactose and glucose to Gal308 were also determined. The enzyme displayed a very high tolerance of galactose and glucose, with the inhibition constants *K*_i,gal_ of 238 mM and *K*_i,glu_ of 1725 mM. In addition, the effects of galactose and glucose on enzymatic activity were investigated at various concentrations of galactose and glucose (Figure 4). Comparing to the inhibition of galactose to other β-galactosidases reported previously, the inhibition of galactose to Gal308 is less pronounced, and the relative activity of Gal308 still reached to 32.5% at a concentration of 400 mM galactose. On the other hand, glucose had an almost negligible inhibitory effect with 89.6% of the initial activity remaining at a concentration of 400 mM glucose.

To investigate the lactose hydrolysis activity of Gal308, an experiment on lactose hydrolysis in milk was performed. After 30 min of incubation at 65°C, 66.5% of milk lactose was hydrolyzed by Gal308 and 31.2% of milk lactose was hydrolyzed by the commercial enzyme. When the incubation time of Gal308 was extended to 45 min, 60 min, the hydrolysis rate of lactose in milk was increased to 82.8% and 93.6%, respectively. Compared to Gal308, the hydrolysis rate for lactose of the commercial enzyme was increased to 45.6% and 51.2% when incubation for 45 min and 60 min, respectively. These results suggest that Gal308 has a better potential for enzyme application in low-lactose milk production than the commercial β-galactosidases.

## Discussion

Until now, the majority of biomolecules obtained via metagenomic strategy are screened from metagenomic libraries constructed from temperate soil samples [20]. Nevertheless, extreme environments, such as solfataric hot springs [21], Urania hypersaline basins [22], provide an almost untapped reservoir of novel biomolecules with biotechnologically valuable properties, these environments are thereby an interesting source for novel biocatalysts that are active under extreme conditions [17]. Recently, some metagenomic libraries derived from extreme habitats have been constructed, and most of them were used to mine novel lipases/esterases [21,22]. All these metagenome-derived esterases displayed habitat-specific properties, such as high thermostability [21] or a preference for high hydrostatic pressure and salinity [22]. However, other enzymes except lipases/esterases obtained via metagenomic approach from extreme environments were seldom reported. In the present study, to identify novel thermostable β-galactosidases, a metagenomic library was constructed using soil samples from Turpan Basin of China, which was regarded as the hottest and driest area of China (the land surface temperature reached up to 76°C) Function-driven screening resulted in the identification of a novel β-galactosidase with a temperature optimum of 78°C and high thermostability. To the best of our knowledge, it is the first report on thermostable β-galactosidase obtained via metagenomic strategy up to now, and it is also the first report of β-galactosidase screened from unculturable microbes of extreme environments. Therefore, this study will enrich the source of β-galactosidases, and attract some attentions to β-galactosidases from extreme habitats and metagenomic library, and thus has some significance to strengthen the theoretical and application research of β-galactosidases from unculturable microbes.

In addition, a comparison of enzymatic properties of Gal308 to other known thermostable β-galactosidases was also performed, and the result was shown in Table 3. Gal308 displayed higher optimal temperature than several thermostable β-galactosidases, including BgaB [8], β-galactosidase from *Rhizomcor* sp. [11], Bgly [12], and β-galactosidase from *Bacillus coagulans* RCS3 [23]. Though some thermostable β-galactosidases had higher optimal temperature than Gal308, their relative enzymatic activities at 65°C (milk pasteurization temperature) were much lower than that of Gal308 (such as β-galactosidases from *Sterigmatomyces elviae* CBS8119 [9] and *Caldicellulosiruptor saccharolyticus *[13]), or were not investigated (such as β-galactosidase from *Pyrococcus woesei *[24] and BgaA from *Thermus* sp. IB-21 [25]). Furthermore, the pH of natural milk is about 6.7-6.8, and thus an ideal β-galactosidase should be optimally active at pH 6.7-6.8. Gal308 displayed a more suitable pH optimum (its pH optimum was 6.8) than several thermostable β-galactosidases such as β-galactosidase from *S. elviae* CGS8119 (its pH optimum was 4.5-5.5) [9], β-galactosidase from *Rhizomucor* sp*.* (its pH optimum was 4.5) [11], and BgaA from *Thermus* sp. IB-21 (its pH optimum was 5.0-6.0) [25]. Considering both of the relative activity at 65°C and optimal pH, only a thermostable β-galactosidase from *Bacillus stearothermophilus *[8] had similar enzymatic properties (80% relative activity at 65°C and a pH optimum of 7.0) with Gal308 among nine known thermostable β-galactosidases. However, the specific activity of the enzyme (5.8 U/mg for ONPG) was much lower than that of Gal308 (185 U/mg for ONPG), and lactose and galactose had a strong competitive inhibition effect against its activity. In addition, lactose is the natural substrate of β-galactosidase, and the higher enzymatic activity for lactose indicates the higher application potential in the food industry. Gal308 displayed a high enzymatic activity (47.6 U/mg) for lactose, which was higher than that of previously described thermostable β-galactosidases, including BgaB (8.5 U/mg) [8], BgaA (36.8 U/mg) from *Thermus* sp. IB-21 [25], and β-galactosidase (13 U/mg) of *Thermus* sp. T2 [26]. However, the activity of Gal308 for lactose was still far less than that for its synthetic substrate-ONPG (185 U/mg). Similar substrate specificity had been observed in several β-galactosidase of GH 42 family, such as a thermostable β-galactosidase from *C. saccharolyticus *[13], a metagenome-derived β-galactosidase [18], and a β-galactosidase from *Alicyclobacillus acidocaldarius*[27]. The results suggested that β-galactosidase from GH42 family had higher catalytic efficiency for ONPG than that for lactose. The direct evolution work of improving the specific activity of Gal308 towards lactose is now under study in this laboratory to obtain a more satisfying β-galactosidase for hydrolysis of lactose in milk.

**Table 3:** The comparison of pH and temperature properties of Gal308 to other known thermostable β-galactosidases

**β-Galactosidase and its origin**	**Substrate**	**Optimal pH**	**Optimal temperature**	**Relative activity**	**Reference**
β-Galactosidase (*T. maritima*)	lactose	6.5	80°C	NT	[7]
BgaB (*B.stearothermophilus*)	ONPG	7.0	70°C	80% (65°C)	[8]
β-Galactosidase (*S. elviae* CBS8119)	ONPG	4.5-5.5	85°C	~45% (65°C)	[9]
β-Galactosidase (*Rhizomucor* sp.)	pNPG	4.5	60°C	NT	[11]
Bgly (*A. acidocaldarius*)	ONPG	5.8	70°C	~85% (65°C)	[12]
β-Galactosidase (*C. saccharolyticus*)	pNPG	6.0	80°C	60% (65°C)	[13]
β-Galactosidase (*B. coagulans* RCS3)	ONPG	6.8	50°C	~40% (60°C)	[23]
β-Galactosidase (*P. woesei*)	ONPG	6.6	90°C	NT	[24]
BgaA (*Thermus* sp. IB-21)	pNPG	5.0-6.0	90°C	90% (95°C)	[25]
Gal308 (uncultured microbes)	lactose	6.8	78°C	87.2% (65°C)	This study

Finally, the reaction products inhibition by galactose and glucose is a common characteristic of β-galactosidase that limits their use in low-lactose milk production. The activity of commercially available β-galactosidase from *Kluyveromyces lactis* is inhibited by galactose with a *K*_i_ of 42 mM [28], and most microbial β-galactosidases reported previously are also strongly inhibited by galactose with *K*_i_ values of 3–45 mM, such as β-galactosidases from *Arthrobacter* sp. [29] and *Hymenaea courbaril *[30], although a β-glactosidase from *Lactobacillus reuteri *[15] with high galactose-tolerance have been identified (*K*_i,gal_ = 115 mM) (Table 4). Furthermore, glucose exhibited strong inhibition to some β-galactosidases like β-galactosidase from *Thermus* sp. T2 [10] and β-galactosidase from *S. solfataricus *[31]. However, the inhibition of glucose to other β-galactosidases is less pronounced [14,15], even a β-galactosidase from *C. saccharolyticus* displayed high glucose-tolerance with a *K*_i_ value of 1170 mM [13]. In this study, the inhibition constant of galactose for Gal308 was 238 mM, which is about 2-fold that for β-galactosidase from *L. reuteri* (115 mM). On the other hand, the inhibition constant of glucose for Gal308 was reached up to 1725 mM, which had been the highest reported inhibition constant for a β-galactosidase to date. Gal308 with high tolerance to glucose and galactose could relieve the inhibition caused by the accumulation of glucose and galactose during the hydrolysis process of lactose, and thus improve its enzymatic activity and hydrolysis efficiency of lactose. The feature of high tolerance to galactose and glucose makes Gal308 have obvious advantage in low-lactose milk production than those commercial β-galactosidases which were sensitive to galactose.

**Table 4:** **Inhibition types and inhibitor constants (*K***_**i**_**) of several β-galactosidases**

**Enzyme source**	**Substrate**	**Inhibitor**	**Inhibition type**	***K***_**i **_**(mM)**	**Reference**
*Thermus* sp. T2	ONPG	Galactose	Competitive	3	[10]
		Glucose	Noncompetitive	50	
*C. saccharolyticus*	pNPG	Galactose	Noncompetitive	12	[13]
		Glucose	Noncompetitive	1170	
*K. lactis*	ONPG	Galactose	Competitive	45	[14]
		Glucose	Noncompetitive	758	
*L. reuteri*	ONPG	Galactose	Competitive	115	[15]
		Glucose	Competitive	683	
*Arthrobacter* sp.	ONPG	Galactose	Competitive	12	[29]
*H. courbaril*	pNPG	Galactose	Competitive	4	[30]
*S. solfataricus*	ONPG	Glucose	Competitive	96	[31]
Unculturable microbes	ONPG	Galactose	Competitive	238	This study
		Glucose	Competitive	1725	

## Conclusion

This work isolated a novel thermostable β-galactosidase (Gal308) from extreme environment, and the recombinant Gal308 with N-terminal fusion tag displayed several novel enzymatic properties, especially high thermostability and tolerance of galactose and glucose. The new enzyme represents a good candidate for the production of low-lactose milk and dairy products. Furthermore, the identification of novel thermostable β-galactosidase from soil samples of Turpan Basin in China highlights the utility of metagenomic approach in discovering potential novel biocatalysts from extreme environments.

## Methods

### Strains, plasmids, and media

*E. coli* DH5α (TaKaRa, Dalian, China) was used as a host for recombinant plasmids. The plasmid pUC19 (TaKaRa) deleted *lacZ* gene was used to construct metagenomic library in this study. To delete *lacZ* gene from pUC19, pUC19 was digested with *Nde*I and *Eco*RI, and a DNA fragment about 2.5 kb was produced. Then two ends of the DNA fragment were ligated together through blunt end ligation, and the plasmid pUC19 with *lacZ* gene deletion was formed. The pET-32a (+) (Novagen, Madison, WI, USA) was used as an overexpression vector to produce the target protein. *E. coli* BL21 (DE3; Novagen) was used as the host for expression of *gal308* gene under the control of the T7 promoter. *E. coli* transformants were grown at 37°C in Luria-Bertani (LB) broth, and the LB medium was supplemented 100 μg/ml ampicillin.

### Materials and chemicals

Lactose and nine chromogenic nitrophenyl analogues, including *o*-nitrophenyl-β-D-galactopyranoside (ONPG), *p*-nitrophenyl-β-D-galactoside, *o*-nitrophenyl-β-D-fucopyranoside, *p*-nitrophenyl-β-D-mannoside, *o*-nitrophenyl-β-D-glucoside, *p*-nitrophenyl-β-D-xyloside, *p*-nitrophenyl-β-D-cellobioside, *p*-nitrophenyl-β-D-lactoside, *p*-nitrophenyl-α-D-galactoside were purchased from Sigma-Aldrich (St. Louis, MO, USA). Restriction endonuleases, T4 DNA ligase, PrimeSTAR HS DNA polymerase were obtained from TaKaRa.

### Conventional DNA manipulation

Conventional DNA manipulations were carried out according to standard techniques or manufacturer’s recommendations. Plasmids were prepared from *E. coli* by using a QIAprep Spin Miniprep Kit according to the manufacturer’s instructions (QIAGEN, Hilden, Germany). DNA fragments were isolated from agarose gels by using a QIAquick Gel Extraction Kit (QIAGEN). Electroporation was performed with a Gene-Pulser II electroporation apparatus (Bio-Rad, Hercules, CA, USA).

### Construction of metagenomic library and screening for β-galactosidase genes

The topsoil samples (5–10 cm depth) were collected from the Mountain of Flames (42° 53^′^ 44^″^ N, 89° 38^′^ 3^″^ E) of the Turpan Basin, Xinjiang province of China. Samples were stored at -80°C until the DNA extraction was performed. Extraction of the total genomic DNA from soil samples was performed using FastDNA Spin Kit for Soil (MP Biomedicals, Santa Ana, CA, USA). Then, Genomic DNA was partially digested with *Bam*HI, and DNA fragments of 2.5-7.5 kb were purified using a QIAquick Gel Extraction Kit and inserted into the pUC19-lacZ-deletion vector, which had been previously digested with *Bam*HI and dephosphorylated with calf intestine alkaline phosphatase (CIAP). Next, *E. coli* DH5α was transformed via electroporation with the library and plated onto LB agar plates containing 100 μg/mL ampicillin, 0.04 mg/mL 5-bromo-4-chloro-3-indolyl-β-D-galactopyranoside (X-Gal) and 0.02 mg/mL isopropyl-β-D-thiogalactopyranoside (IPTG). A functional β-galactosidase screening was visualized performed by blue color, which was resulted from the hydrolysis of X-Gal. Finally, plasmid DNA of positive clones was extracted and sequenced on ABI 377 DNA sequencer.

### Analysis of β-galactosidase gene

The open reading frame search from DNA sequences was carried out using ORF-finder (NCBI) (http://www.ncbi.nlm.nih.gov/), and database homology search was performed with BLAST program provided by NCBI. Furthermore, the multiple amino acid sequence alignment of Gal308 and known homologous β-galactosidases and the analysis of conserved amino acid residues and active site residues of Gal308 were performed by using ClustalW2 program (http://www.ebi.ac.uk/Tools/msa/clustalw2/).

### Expression and purification of recombinant protein

The PCR primers for *gal308* amplification were listed as follows: gal308-f, 5′-CGC*GGATCC*ATGGCCTTTCCAAACGAGCATGGAG, in which the *Bam*HI site was shown in italics; gal308-r, 5′-CCC*AAGCTT*TCCCTCGTGTTCTTCATAGAC, in which the *Hin*dIII site was shown in italics. PCR reaction conditions were: 98°C, 10 sec (denaturation); 68°C, 3 min (annealing and extension); repeated for 30 cycles. The PCR product was digested with *Bam*HI/*Hin*dIII and subcloned to *Bam*HI/*Hin*dIII-treated expression vector pET-32a (+) with a six-histidine tag for purification. The recombinant vector was transformed into *E. coli* BL21 (DE3), and then the cells were plated on LB agar containing 100 μg/ml ampicillin. The transformant was grown in a 100-ml flask containing 10 ml LB medium supplemented with 100 μg/ml ampicillin at 37°C until the optical density at 600 nm reached to 1.0, and then IPTG was added to final concentration of 1.2 mM, and the culture was incubated at 30°C for 8 h with shaking at 200 rpm. Cells were then collected by centrifugation (6,000 g for 20 min at 4°C) and stored at -20°C for later purification. All purification steps were performed according to the instruction of His Bind Purification Kit (Novagen). In brief, the cells were suspended in binding buffer (0.5 M NaCl, 5 mM imidazole, 20 mM Tris–HCl, pH 7.9) followed by sonication on ice. The supernatant was collected by centrifugation at 14,000 g for 20 min at 4°C, and then they were loaded onto a Ni-NTA His · Bind column (Novagen) pre-equilibrated with binding buffer. The column was washed with binding buffer and washing buffer (0.5 M NaCl, 60 mM imidazole, 20 mM Tris–HCl, pH 7.9). Finally, the bound protein was eluted with eluting buffer (1 M imidazole, 0.5 M NaCl, 20 mM Tris–HCl, pH 7.9). Next, the purified enzyme in elution buffer was collected and further removed imidazole by dialysis before the characterization of the enzyme. The dialysis was performed three times, and each dialysis lasted for two hours in dialysis buffer (100 mM NaCl, 3 mM dithiothreitol, 20 mM Tris–HCl, pH 7.9).

### Determination of molecular mass

The molecular mass of the denatured protein was determined by sodium dodecyl sulfate-polyacrylamide gel electrophoresis (SDS-PAGE). Proteins were stained with Coomassie brilliant blue G-250. The molecular mass of the enzyme subunit was estimated using protein marker (Tiangen Biotech, Beijing, China) as standard.

### Analysis of enzyme activity

The β-galactosidase activity was measured using two substrates including ONPG and lactose in this study. The β-galactosidase activity for ONPG was measured by following the amount *o*-nitrophenol released from ONPG. The reaction mixture was composed of 100 μL of the enzyme solution and 400 μL of ONPG solution (2.5 g/L in 100 mM Tris–HCl buffer at pH 6.8). After incubation at 78°C for 15 min, the reaction was terminated by adding an equal volume of 1.0 M Na_2_CO_3_. The released *o*-nitrophenol was quantitatively determined by measuring at *A*_*405*_. One unit of activity was defined as the amount of enzyme needed to produce 1 μmol of *o*-nitrophenol per minute under the assay condition. The specific activity was expressed as units per milligram of protein. Assays for activity towards lactose were performed in the same buffer containing 100 μl of enzyme solution and 5% lactose, and the reaction was stopped by boiling for 10 min, and the concentration of glucose was determined using a glucose oxidase-peroxidase assay kit (Sigma-Aldrich). The released glucose was quantitatively determined by measuring *A*_*492*_. One unit of enzyme activity was defined as the amount of activity required to release 1 μmol of glucose per minute.

### Effect of pH and temperature on enzyme activity

The optimal pH of the enzyme was measured using lactose as a substrate at 78°C and a pH range of 2.0 - 10.0. The buffers used for the measurement were as below: 0.1 M disodium hydrogen phosphate-citrate buffer (pH 2.0 - 5.0), 0.1 M potassium phosphate buffer (pH 6.0 - 8.0), and 0.1 M glycine - sodium hydroxide buffer (pH 9.0 - 10.0). The pH stability was investigated under standard assay conditions after incubation of the purified enzyme for 24 h at 4°C in the above buffer systems in the absence of substrate. In the same way, the temperature optimum was also determined by measuring enzymatic activity at pH 6.8 in the temperature range of 40°C - 90°C (40°C, 50°C, 60°C, 65°C, 70°C, 75°C, 80°C, 85°C, 90°C). Temperature stability was measured by analyzing residual activity after incubation of aliquots of enzyme for 1 h at different temperatures.

### Effect of metal ions on enzyme activity

The metal ions for test were 1 mM of CaCl_2_, CuSO_4_, NaCl, KCl, FeCl_3_, AlCl_3_, MgCl_2_, MnCl_2_, and ZnCl_2_. After pre-incubating the enzyme solutions containing each individual metal ion in 100 mM Tris–HCl buffer (pH 6.8) at 4°C for 15 min, the natural substrate lactose was then added, and the enzyme activity was measured under standard conditions. A control without metal ion was also performed. The amount of enzymatic activity was calculated as a percentage of the activity comparing to that of the control.

### Determination of substrate specificity and kinetic parameters

Substrate specificity of Gal308 against lactose and nine different chromogenic nitrophenyl analogues was determined by incubating the enzyme at 78°C for 5 min in 100 mM Tris–HCl buffer (pH 6.8) containing 5 mM final concentration of lactose or nitrophenyl substrate. The kinetic parameters (*K*_m_ and *k*_cat_) for the recombinant enzyme were investigated by assaying the enzymatic activity in 0.1 M phosphate buffered saline (PBS, 0.1 M NaH_2_PO_4_, 0.1 M Na_2_HPO_4_, 0.1 M NaCl, pH 6.8) at 78°C with two substrates, ONPG and lactose. All kinetic studies were performed three times, and kinetic data were fitted to hyperbola by using the Michaelis-Menton equation. Kinetic analyses by curve fitting were performed with the SigmaPlot software (Systat Software, Chicago, IL, USA). Furthermore, Lineweaver-Burk plots (1/V vs. 1/[S]) were used to investigate the inhibition type of galactose and glucose on the enzymatic activity. The inhibition constants (*K*_i_ values) of galactose and glucose to Gal308 were obtained by fitting to Cornish-Bowden plot using various concentrations of galactose (0 – 20 mM) and glucose (0 – 400 mM) with various concentrations of ONPG (0.05 - 1 mM) as a substrate [32].

### Effects of galactose and glucose on the enzyme activity

The effects of galactose and glucose on the activity of Gal308 were determined at the concentrations of galactose from 25 to 400 g/L and glucose from 50 to 400 g/L using ONPG as substrate [13]. The relative activity was defined as the relative value to the maximum activity without galactose or glucose.

### Hydrolysis of lactose in milk

Milk containing 5% (w/v) lactose was added with equal amount of enzyme (20 U for 1 g of lactose) including recombinant Gal308 or a commercial product of β-galacosidase (Maxilact, DSM China, Shanghai, China), and the solutions were incubated for 30 min, 45 min, and 60 min with shaking (150 rpm) at 65°C, respectively. Then, mixed the aliquots of the digest with the same volume of 10% trichloroacetic acid solution and centrifuged, and adjusted pH of the supernatant to 7.0 with NaOH immediately. Finally, a commercial enzymatic test kit (Sunbio, Beijing, China) was used to test the concentration of glucose liberated by the enzyme, and glucose concentration was determined based on *A*_*530*_ measurements of the dye produced by oxidation of a chromogen (4-aminopyrine).

### Nucleotide sequence accession number

The nucleotide sequence data reported here have been submitted to the nucleotide sequence databases (GenBank) under accession number (JQ009372).

## Abbreviations

ONPG: *O*-nitrophenyl-β-D-galactopyranoside; pNPG: *p*-Nitrophenyl-β-D-galactopyranoside; NT: Not tested.

## Competing interests

The authors declared that they have no competing interests.

## Authors’ contributions

XZ: performed construction of metagenomic library and gene cloning. HL: performed gene expression in *E. coli* and enzyme characterization. CJL: extracted DNA from soil samples. TM: collected soil samples of Turpan Basin. GL: designed and supervised the experiment, drafted and revised the manuscript. YHL conceived this study. All authors have read and approved the manuscript.
